# Supplementary material for: Effectiveness of roadside alcohol testing in reducing fatal accidents and fatal drinking-driving accidents: A multi-city study in China
Source: PLoS One. 2026 Jan 23;21(1):e0338886. doi: 10.1371/journal.pone.0338886 (PMC12829829; doi:10.1371/journal.pone.0338886)
Supplement: S1 File — (ZIP) [file pone.0338886.s001.zip › supple_code.docx]

#Since both the court judgment documents and the geographic shapefiles are based on Chinese-language text, the text mining code necessarily relies on Chinese characters for pattern matching. Key legal phrases such as “本院认为” ("this court believes") and outcome indicators like “人死亡” ("person(s) died") appear only in Chinese. Therefore, while the code structure (e.g., logic, data handling, modeling) follows standard Python practices, the regular expressions and text processing components must remain in Chinese. A full conversion to English is not feasible without first translating the source data, which would risk losing the legal and linguistic nuances essential for accurate extraction.

# #TEXT MINING

## #Extration of location

import csv

import pandas as pd

import numpy as np

from tqdm import tqdm

import geopandas as gpd

import re

file_path = r"YOUR_PATH\raw_data.csv" #Court files were collected from [https://www.macrodatas.cn](https://www.macrodatas.cn/), using the keywords “危险驾驶” (dangerous driving) and “交通肇事” (traffic accident crime) to filter court records by “案由”(case type). All traffic-related crimes matching these criteria were included.

df = pd.read_csv(file_path)

print(df.head())

df.columns

folder_path = r"YOUR_PATH\2019中国地图-审图号GS(2019)1822号" #map could be retrieved from (http://bzdt.ch.mnr.gov.cn/)

city_shp = "市（等积投影）.shp"

county_shp = "县（等积投影）.shp"

city_gdf = gpd.read_file(folder_path + "\\" + city_shp)

print(city_gdf.head())

county_gdf = gpd.read_file(folder_path + "\\" + county_shp)

print(county_gdf.head())

city_gdf

county_gdf

unique_courts = df['法院'].dropna().unique()

court_to_city = {}

for court in tqdm(unique_courts, desc="匹配法院与城市"):

found = False

for _, row in county_gdf.iterrows():

name = row['NAME']

city = row['市']

if pd.isna(name) or pd.isna(city):

continue

if name in court:

court_to_city[court] = city

found = True

break

if not found:

court_to_city[court] = np.nan

df['city'] = df['法院'].map(court_to_city)

unique_courts = df['法院'].dropna().unique()

unique_cities = county_gdf['市'].dropna().unique()

court_to_city = {}

for court in tqdm(unique_courts, desc="匹配法院与市"):

found = False

for city_name in unique_cities:

if city_name in court:

court_to_city[court] = city_name

found = True

break

if not found:

court_to_city[court] = np.nan

df['city'] = df.apply(lambda row: row['city'] if pd.notna(row['city']) else court_to_city.get(row['法院'], np.nan), axis=1)

def clean_region_name(name):

return re.sub(r'(市|自治区|特别行政区|地区|盟)$', '', str(name))

county_gdf = county_gdf.copy()

county_gdf['市_clean'] = county_gdf['市'].apply(clean_region_name)

if 'city' not in df.columns:

df['city'] = np.nan

unmatched_courts = df.loc[df['city'].isna(), '法院'].dropna().unique()

court_to_city = {}

for court in tqdm(unmatched_courts, desc="匹配未填的法院与市"):

found = False

for _, row in county_gdf.iterrows():

clean_keyword = row['市_clean']

original_city_name = row['市']

if clean_keyword in court:

court_to_city[court] = original_city_name

found = True

break

if not found:

court_to_city[court] = np.nan

df.loc[df['city'].isna(), 'city'] = df.loc[df['city'].isna(), '法院'].map(court_to_city)

## #Extration of time

import pandas as pd

import re

pattern = r"(如不服本判决|附法律依据及法律条文).*"

df['全文'] = df['全文'].apply(lambda x: re.sub(pattern, "", str(x)))

pattern1 = r"(如不服本判决|附法律依据及法律条文)"

contains_pattern1 = df['全文'].str.contains(pattern1, regex=True)

print(df[contains_pattern1])

df.rename(columns={'全文': 'Content'}, inplace=True)

def extract_closest_date_after(text, keywords=["指控", "经审理查明"]):

date_pattern = r'\d{4}年\d{1,2}月\d{1,2}日'

dates = [(m.start(), m.group()) for m in re.finditer(date_pattern, text)]

if not dates:

return 'na'

keyword_positions = [m.end() for keyword in keywords for m in re.finditer(keyword, text)]

if not keyword_positions:

return 'na'

keyword_positions.sort(reverse=True)

for position in keyword_positions:

for start, date in dates:

if position < start <= position + 15:

return date

return 'na'

df['Content'] = df['Content'].astype(str)

df['Occur_date'] = df['Content'].apply(extract_closest_date_after)

df['Occur_date'] = pd.to_datetime(df['Occur_date'], errors='coerce')

df['year'] = df['Occur_date'].dt.year

df['year_month'] = df['Occur_date'].dt.to_period('M').astype(str)

## #Extraction of DUI

import numpy as np

import re

df.loc[df['Content'].str.contains('酒驾|系酒驾|系饮酒驾驶|系醉酒驾驶|为酒驾|为饮酒驾驶|为醉酒驾驶', na=False), 'alcohol'] = 1

criminal_history_terms = r'酒精|乙醇'

crime_types_terms = r'mg/100ml|毫克每毫升|毫克每100毫升|mg/ml|mg/mL|毫克'

df.loc[

df['Content'].str.contains(criminal_history_terms, na=False) &

df['Content'].str.contains(crime_types_terms, na=False),

'alcohol'

] = 1

df.loc[

df['Content'].str.contains(

'非酒驾|嫌疑人未饮酒|嫌疑人未检出酒精|嫌疑人未测出酒精|无酒驾|嫌疑人未检出乙醇|嫌疑人酒精浓度为0mg/100ml|嫌疑人血液酒精浓度为0mg/100ml',

na=False

),

'alcohol'

] = 0

df['alcohol'] = df['alcohol'].fillna(0)

df['alcohol_accident_count'] = (

(df['alcohol'].fillna(0) == 1) &

(df['案由'].fillna('') == '危险驾驶')

).astype(int)

## #Extraction of mortality number

import numpy as np

import re

import cn2an

def extract_mortality_count(row):

text = row['Content']

mortality = row['mortality']

pattern = r'本院认为.*?([一二三四五六七八九十百千万0-9]+)(?=人死亡)'

match = re.search(pattern, text)

if match:

num_str = match.group(1)

try:

return cn2an.cn2an(num_str, 'smart')

except:

try:

return int(num_str)

except:

return 1

else:

if '本院认为' in text and '死亡' in text:

return 1

else:

if mortality == 1:

return 1

else:

return np.nan

df['mortality_count'] = df.apply(extract_mortality_count, axis=1)

valid_mortality = df['mortality_count'].dropna()

total = valid_mortality.sum()

mean = valid_mortality.mean()

min_val = valid_mortality.min()

max_val = valid_mortality.max()

df['year'] = df['裁判日期'].dt.year

df_filtered = df[(df['year'] >= 2014) & (df['year'] <= 2020)]

result = df_filtered.groupby(['year_month', 'city'])['mortality_count'].sum().reset_index()

condition = (df_filtered['案由'] == ‘危险驾驶’) & (df_filtered['alcohol'] == 1)

conditional_counts = df_filtered[condition].groupby(['year_month', 'city']).size().reset_index(name='alcohol_accident_count')

result = result.merge(conditional_counts, on=['year_month', 'city'], how='left')

result['alcohol_accident_count'] = result['alcohol_accident_count'].fillna(0).astype(int)

alcohol_df = df_filtered[df_filtered['alcohol'] == 1]

alcohol_mortality = (

alcohol_df

.groupby(['year_month', 'city'])['mortality_count']

.sum()

.reset_index(name='mortality_count_alcohol')

)

result = result.merge(

alcohol_mortality,

on=['year_month', 'city'],

how='left'

)

result['mortality_count_alcohol'] = (

result['mortality_count_alcohol']

.fillna(0)

.astype(int)

)

result['year'] = result['year_month'].dt.year

city_year_stats = result.groupby(['city', 'year'])[['mortality_count', 'alcohol_accident_count']].agg(

lambda x: (x == 0).all()

).reset_index()

city_year_to_drop = city_year_stats[

(city_year_stats['mortality_count'] == True) | (city_year_stats['alcohol_accident_count'] == True)

]['city'].unique()

result_filtered = result[~result['city'].isin(city_year_to_drop)].copy()

total_sums = result_filtered[['mortality_count', 'alcohol_accident_count', 'mortality_count_alcohol']].sum()

print(total_sums)

total_sums

df=result_filtered.copy()

# #CIYT-LEVEL DATA PREPARATION

## #Population preparation

import csv

import pandas as pd

import numpy as np

from tqdm import tqdm

popu_path = r"YOUR_PATH\population.csv" #Population data can be retrieved from the sixth census and seventh census

df_long = pd.read_csv(popu_path)

df_long = pd.melt(

df_trimmed,

id_vars=['省市名称'],

var_name='年份',

value_name='人口'

)

df_long['年份'] = df_long['年份'].astype(int)

df_2020_over_5million = df_long[(df_long['年份'] == 2020) & (df_long['人口'] > 500)]

df_2020_1_to_5million = df_long[(df_long['年份'] == 2020) & (df_long['人口'] <= 500)]

five_million_cities = df_2020_over_5million['省市名称'].tolist()

one_to_five_million_cities = df_2020_1_to_5million['省市名称'].tolist()

epsilon = 1e-6

cols = ['mortality_count', 'alcohol_accident_count', 'mortality_count_alcohol']

for col in cols:

df[col] = df[col].fillna(epsilon)

df[col] = df[col].replace(0, epsilon)

df = df.rename(columns={

'year': 'Occur_year',

'alcohol_accident_count': 'roadside_check'

})

df_long['年份'] = df_long['年份'].astype(int)

df['Occur_year'] = df['Occur_year'].astype(int)

merged_df = pd.merge(df_long, df, left_on=['省市名称', '年份'], right_on=['city', 'Occur_year'], how='outer', indicator=True)

merged_success = merged_df[merged_df['_merge'] == 'both']

merged_fail = merged_df[merged_df['_merge'] != 'both']

successcity=merged_success['city'].unique()

len(successcity)

target_cities = set(five_million_cities) | set(one_to_five_million_cities)

merged_df = merged_df[merged_df['省市名称'].isin(target_cities)]

## #City-level confounders

file_path = r"D:\Your_path\city_parameters.xlsx" # data can be retrieved from municipal-level statistical bureaus

df_confound = pd.read_excel(file_path)

df_confound

df_confound.rename(columns={'城市': 'city', '年份': 'Occur_year'}, inplace=True)

df_merged = pd.merge(merged_df, df_confound, on=['city', 'Occur_year'], how='left')

df_merged.drop(columns=['市级行政代码', '省级行政代码', '省份'], inplace=True)

merged_cities = df_merged['city'].unique()

confound_cities = df_confound['city'].unique()

remaining_cities = df_merged_filtered['city'].nunique()

unique_cities = df_merged_filtered['city'].unique()

valid_columns = []

invalid_columns = []

cols_with_nan = [col for col in valid_columns if df_merged_filtered[col].isnull().any()]

df_interpolated = (

df_merged_filtered

.groupby('city', group_keys=False)

.apply(lambda group: group.sort_values('Occur_year').interpolate(method='linear', limit_direction='both'))

)

nan_summary = df_interpolated[cols_with_nan].isnull().sum()

remaining_nans = nan_summary[nan_summary > 0]

nan_summary = df_interpolated.isnull().sum()

cols_to_drop = nan_summary[nan_summary > 0].index.tolist()

df_interpolated_cleaned = df_interpolated.drop(columns=cols_to_drop)

missing_cols = df_interpolated_cleaned.isnull().sum()

missing_cols = missing_cols[missing_cols > 0]

df_loaded = df_interpolated_cleaned.copy()

print(list(df_loaded.columns))

columns_to_keep = [

'人口', 'year_month',

'Occur_year',

'city',

'mortality_count',

'roadside_check',

'mortality_count_alcohol',

'每10万人地区生产总值_当年价格-全市（亿元）',

'每10万人公路客运量（万人）全市',

'每10万人地方一般公共预算收入（万元）全市',

'每10万人医院床位数（张）全市'

]

df_loaded = df_loaded[columns_to_keep]

df_loaded['mortality_rate'] = df_loaded['mortality_count'] / (df_loaded['人口'] * 10)

df_loaded['roadside_check_rate'] = df_loaded['roadside_check'] / (df_loaded['人口'] * 10)

df_loaded['alcohol_mortality_rate'] = df_loaded['mortality_count_alcohol'] / (df_loaded['人口'] * 10)

df_loaded = df_loaded.drop(columns=[

'mortality_count',

'roadside_check',

'mortality_count_alcohol'

])

column_rename_map = {

'人口': 'population_10,000',

'Occur_year': 'occur_year',

'city': 'city',

'每10万人地区生产总值_当年价格-全市（亿元）': 'gdp(100,000,000)_per_100k',

'每10万人公路客运量（万人）全市': 'passenger_volume(10,000)_per_100k',

'每10万人地方一般公共预算收入（万元）全市': 'fiscal_income(10,000)_per_100k',

'每10万人医院床位数（张）全市': 'hospital_beds_per_100k',

'mortality_rate': 'mortality_rate',

'roadside_check_rate': 'roadside_check_rate',

'alcohol_mortality_rate': 'alcohol_mortality_rate'

}

df_loaded = df_loaded.rename(columns=column_rename_map)

df_loaded['city_size'] = 0

cities_large = df_loaded.loc[

(df_loaded['occur_year'] == 2020) & (df_loaded['population_10,000'] >= 500),

'city'

].unique()

df_loaded['city_size'] = df_loaded['city'].isin(cities_large).astype(int)

df_loaded = df_loaded[

(df_loaded['occur_year'] >= 2014) &

(df_loaded['occur_year'] <= 2020)

]

df_loaded['percentage_alcohol_mortality'] = df_loaded['alcohol_mortality_rate'] / df_loaded['mortality_rate']

save_path = r"YOUR_PATH\supple_df.csv"

df_loaded.to_csv(save_path, index=False, encoding='utf-8-sig') #The dataset supple_df.csv can be found in supplement files

# #DATA ANALYSIS

## #IRF Analysis

import pandas as pd

import numpy as np

from statsmodels.tsa.stattools import adfuller, grangercausalitytests

from statsmodels.tsa.api import VAR

from statsmodels.tsa.statespace.sarimax import SARIMAX

import matplotlib.pyplot as plt

import seaborn as sns

from matplotlib import font_manager

save_path = r"YOUR_PATH\supple_df.csv" #The dataset supple_df.csv can be found in supplement files

df_loaded = pd.read_csv(save_path)

df_loaded['year_month'] = pd.to_datetime(df_loaded['year_month'])

df_small = df_loaded[df_loaded['city_size'] == 0].copy()

df_large = df_loaded[df_loaded['city_size'] == 1].copy()

def adf_test(series, name):

result = adfuller(series.dropna())

print(f"ADF检验 - {name}：p-value={result[1]:.4f} | {'非平稳' if result[1]>0.05 else '平稳'}")

print("\n小城市：")

adf_test(df_small['roadside_check_rate'], "检查率")

adf_test(df_small['alcohol_mortality_rate'], "酒驾死亡率")

adf_test(df_small['mortality_rate'], "死亡率")

print("\n大城市：")

adf_test(df_large['roadside_check_rate'], "检查率")

adf_test(df_large['alcohol_mortality_rate'], "酒驾死亡率")

adf_test(df_large['mortality_rate'], "死亡率")

prop_en = font_manager.FontProperties(family='Times New Roman')

def run_var_irf_subplot(df_small, df_large):

fig, axes = plt.subplots(2, 2, figsize=(14, 10), constrained_layout=True)

plt.style.use('grayscale')

city_data = {

'Small cities': df_small,

'Large cities': df_large

}

responses = ['mortality_rate', 'alcohol_mortality_rate']

titles = [

"Small cities: Roadside checks → Mortality rate",

"Small cities: Roadside checks → Alcohol-related mortality rate",

"Large cities: Roadside checks → Mortality rate",

"Large cities: Roadside checks → Alcohol-related mortality rate"

]

idx = 0

horizon = 8

for city_name, df_city in city_data.items():

for response in responses:

ax = axes[idx % 2, idx // 2]

data = df_city[['roadside_check_rate', response]].dropna()

model = VAR(data)

lag_sel = model.select_order(12)

lag = lag_sel.aic

results = model.fit(lag)

irf = results.irf(horizon)

irf_values = irf.irfs[:, 1, 0]

stderr = irf.stderr(orth=False)[:, 1, 0]

lower_band = irf_values - 1.96 * stderr

upper_band = irf_values + 1.96 * stderr

x = np.arange(horizon + 1)

ax.plot(x, irf_values, label='IRF', color='black')

ax.fill_between(x, lower_band, upper_band, color='gray', alpha=0.3, label='95% CI')

ax.axhline(0, color='black', linewidth=0.8, linestyle='--')

ax.set_title(titles[idx], fontproperties=prop_en, fontsize=12)

ax.set_xlabel("Lag", fontproperties=prop_en, fontsize=10)

ax.set_ylabel("Response", fontproperties=prop_en, fontsize=10)

ax.tick_params(labelsize=9)

for label in ax.get_xticklabels() + ax.get_yticklabels():

label.set_fontproperties(prop_en)

ax.legend(prop=prop_en, fontsize=9)

ax.grid(False)

idx += 1

plt.suptitle("", fontproperties=prop_en, fontsize=16)

plt.show()

run_var_irf_subplot(df_small, df_large)

def check_var_irf_robustness(df_city, response, city_label, lags=[4, 8], horizon=8):

data = df_city[['roadside_check_rate', response]].dropna()

model = VAR(data)

plt.figure(figsize=(7, 4))

for lag in lags:

results = model.fit(lag)

irf = results.irf(horizon)

irf_values = irf.irfs[:, 1, 0]

plt.plot(np.arange(horizon + 1), irf_values, label=f"Lag = {lag}")

plt.axhline(0, color='black', linestyle='--', linewidth=0.8)

plt.title(f"Robustness check ({city_label}): Roadside checks → {response}",

fontproperties=prop_en, fontsize=12)

plt.xlabel("Lag horizon", fontproperties=prop_en)

plt.ylabel("IRF response", fontproperties=prop_en)

plt.legend(prop=prop_en)

plt.grid(False)

plt.show()

check_var_irf_robustness(df_small, 'mortality_rate', 'Small cities', lags=[4, 8])

check_var_irf_robustness(df_small, 'alcohol_mortality_rate', 'Small cities', lags=[4, 8])

check_var_irf_robustness(df_large, 'mortality_rate', 'Large cities', lags=[4, 8])

check_var_irf_robustness(df_large, 'alcohol_mortality_rate', 'Large cities', lags=[4, 8])

def summarize_irf_results(df_city, response, city_label, lags=[4, 8], horizon=8):

data = df_city[['roadside_check_rate', response]].dropna()

model = VAR(data)

results_list = []

for lag in lags:

results = model.fit(lag)

irf = results.irf(horizon)

irf_values = irf.irfs[:, 1, 0]

peak_val = irf_values.max()

trough_val = irf_values.min()

peak_time = np.argmax(irf_values)

trough_time = np.argmin(irf_values)

end_val = irf_values[-1]

results_list.append({

'City group': city_label,

'Response variable': response,

'Lag order': lag,

'Peak (max)': round(peak_val, 4),

'Peak time': peak_time,

'Trough (min)': round(trough_val, 4),

'Trough time': trough_time,

'Value at horizon (month 8)': round(end_val, 4)

})

return pd.DataFrame(results_list)

df_results = pd.concat([

summarize_irf_results(df_small, 'mortality_rate', 'Small cities', lags=[4, 8]),

summarize_irf_results(df_small, 'alcohol_mortality_rate', 'Small cities', lags=[4, 8]),

summarize_irf_results(df_large, 'mortality_rate', 'Large cities', lags=[4, 8]),

summarize_irf_results(df_large, 'alcohol_mortality_rate', 'Large cities', lags=[4, 8])

], ignore_index=True)

print(df_results)

## #Marginal effects of roadside check rate on mortality outcomes

import pandas as pd

import numpy as np

import matplotlib.pyplot as plt

import seaborn as sns

from xgboost import XGBRegressor

from sklearn.metrics import r2_score, mean_squared_error

from sklearn.model_selection import KFold

from PIL import Image

import os

df_loaded['year_month'] = pd.to_datetime(df_loaded['year_month'])

df_loaded = df_loaded.sort_values(by=['city', 'year_month'])

df_loaded['month'] = df_loaded['year_month'].dt.month

df_loaded['sin_month'] = np.sin(2 * np.pi * df_loaded['month'] / 12)

df_loaded['cos_month'] = np.cos(2 * np.pi * df_loaded['month'] / 12)

periods = {

'2014-2016': (2014, 2016),

'2017-2020': (2017, 2020)

}

for period_name, (start_year, end_year) in periods.items():

df_period = df_loaded[df_loaded['occur_year'].between(start_year, end_year)].copy()

df_period = df_period.sort_values(by=['city', 'year_month'])

for lag in [4, 8]:

df_loaded.loc[df_period.index, f'roadside_check_rate_lag{lag}'] = (

df_period.groupby('city')['roadside_check_rate'].shift(lag)

)

features = ['roadside_check_rate', 'roadside_check_rate_lag4', 'roadside_check_rate_lag8',

'gdp(100,000,000)_per_100k', 'passenger_volume(10,000)_per_100k',

'fiscal_income(10,000)_per_100k', 'hospital_beds_per_100k',

'sin_month', 'cos_month']

targets = ['mortality_rate', 'alcohol_mortality_rate']

target_labels = ['Mortality rate', 'Alcohol mortality rate']

feature_lags = ['roadside_check_rate', 'roadside_check_rate_lag4', 'roadside_check_rate_lag8']

feature_colors = {

'roadside_check_rate': 'black',

'roadside_check_rate_lag4': 'dimgray',

'roadside_check_rate_lag8': 'lightgray'

}

plt.rcParams["font.family"] = "Times New Roman"

plt.style.use('grayscale')

fig, axes = plt.subplots(nrows=2, ncols=2, figsize=(14, 10), constrained_layout=True)

city_groups = {

'Small cities': 0,

'Large cities': 1

}

targets = ['mortality_rate', 'alcohol_mortality_rate']

target_labels = ['Mortality rate', 'Alcohol mortality rate']

feature_lags = ['roadside_check_rate', 'roadside_check_rate_lag4', 'roadside_check_rate_lag8']

feature_colors = {

'roadside_check_rate': 'black',

'roadside_check_rate_lag4': 'dimgray',

'roadside_check_rate_lag8': 'lightgray'

}

features = ['roadside_check_rate', 'roadside_check_rate_lag4', 'roadside_check_rate_lag8',

'gdp(100,000,000)_per_100k', 'passenger_volume(10,000)_per_100k',

'fiscal_income(10,000)_per_100k', 'hospital_beds_per_100k',

'sin_month', 'cos_month']

#Only the machine learning model shown in the manuscript figure was retained for demonstration.

for i, target in enumerate(targets):

for j, (group_name, size_value) in enumerate(city_groups.items()):

ax = axes[i, j]

df_group = df_loaded[df_loaded['city_size'] == size_value].copy()

df_group = df_group.dropna(subset=features + targets)

for var in feature_lags:

low, high = df_plot[var].quantile([0.05, 0.95])

df_plot = df_plot[(df_plot[var] >= low) & (df_plot[var] <= high)]

if df_plot.empty:

ax.set_title(f"{group_name} - {target_labels[i]} (No data)", fontsize=16)

ax.axis('off')

continue

X_all = df_plot[features]

y_all = df_plot[target]

kf = KFold(n_splits=3, shuffle=True, random_state=42)

y_true_all = []

y_pred_all = []

for train_idx, test_idx in kf.split(X_all):

X_train, X_test = X_all.iloc[train_idx], X_all.iloc[test_idx]

y_train, y_test = y_all.iloc[train_idx], y_all.iloc[test_idx]

model = XGBRegressor(n_estimators=100, max_depth=4, learning_rate=0.1, random_state=42)

model.fit(X_train, y_train)

y_pred_all.extend(model.predict(X_test))

y_true_all.extend(y_test)

r2 = r2_score(y_true_all, y_pred_all)

rmse = mean_squared_error(y_true_all, y_pred_all, squared=False)

model = XGBRegressor(n_estimators=100, max_depth=4, learning_rate=0.1, random_state=42)

model.fit(X_all, y_all)

for var in feature_lags:

x = df_plot[[var]].values

x_range = np.linspace(x.min(), x.max(), 100).reshape(-1, 1)

x_input = np.zeros((100, len(feature_lags)))

x_input[:, feature_lags.index(var)] = x_range.ravel()

control_means = df_plot[[f for f in features if f not in feature_lags]].mean().values

full_input = np.hstack([x_input, np.tile(control_means, (100, 1))])

y_pred_curve = model.predict(full_input)

ax.plot(x_range, y_pred_curve, color=feature_colors[var], label=var.replace('_', ' ').capitalize())

ax.grid(False)

ax.set_title(f"{group_name} - {target_labels[i]}\nR²={r2:.3f}, RMSE={rmse:.4f}",

fontsize=16, fontweight='bold')

ax.set_xlabel("Roadside check rate", fontsize=14)

ax.set_ylabel(target_labels[i], fontsize=14)

ax.tick_params(labelsize=12)

if i == 0 and j == 1:

ax.legend(fontsize=12, loc='upper right')

plt.suptitle("XGBoost Marginal Effects", fontsize=18, fontweight='bold')

plt.show()

## #SHAP feature importance with direction

import matplotlib.pyplot as plt

import numpy as np

import pandas as pd

import shap

from xgboost import XGBRegressor

from sklearn.metrics import r2_score, mean_squared_error

from sklearn.model_selection import KFold

plt.rcParams["font.family"] = "Times New Roman"

plt.style.use('grayscale')

fig, axes = plt.subplots(nrows=2, ncols=2, figsize=(14, 10), constrained_layout=True)

city_groups = {

'Small cities': 0,

'Large cities': 1

}

targets = ['mortality_rate', 'alcohol_mortality_rate']

target_labels = ['Mortality rate', 'Alcohol mortality rate']

for i, target in enumerate(targets):

for j, (group_name, size_value) in enumerate(city_groups.items()):

ax = axes[i, j]

df_group = df_loaded[df_loaded['city_size'] == size_value].copy()

df_group = df_group.dropna(subset=features + targets)

df_plot = df_group.copy()

for var in feature_lags:

low, high = df_plot[var].quantile([0.05, 0.95])

df_plot = df_plot[(df_plot[var] >= low) & (df_plot[var] <= high)]

if df_plot.empty:

ax.set_title(f"{group_name} - {target_labels[i]}: No data", fontsize=12, fontname='Times New Roman')

ax.axis('off')

continue

X_all = df_plot[features]

y_all = df_plot[target]

kf = KFold(n_splits=3, shuffle=True, random_state=42)

y_true_all = []

y_pred_all = []

for train_idx, test_idx in kf.split(X_all):

X_train, X_test = X_all.iloc[train_idx], X_all.iloc[test_idx]

y_train, y_test = y_all.iloc[train_idx], y_all.iloc[test_idx]

model = XGBRegressor(n_estimators=100, max_depth=4, learning_rate=0.1, random_state=42)

model.fit(X_train, y_train)

y_pred = model.predict(X_test)

y_pred_all.extend(y_pred)

y_true_all.extend(y_test)

r2 = r2_score(y_true_all, y_pred_all)

rmse = mean_squared_error(y_true_all, y_pred_all, squared=False)

model = XGBRegressor(n_estimators=100, max_depth=4, learning_rate=0.1, random_state=42)

model.fit(X_all, y_all)

explainer = shap.Explainer(model)

shap_values = explainer(X_all)

mean_abs_shap = np.abs(shap_values.values).mean(axis=0)

mean_shap = shap_values.values.mean(axis=0)

importance_df = pd.DataFrame({

'feature': features,

'mean_abs_shap': mean_abs_shap,

'mean_shap': mean_shap,

'direction': ['Positive' if val > 0 else 'Negative' for val in mean_shap]

}).sort_values(by='mean_abs_shap', ascending=True)

for idx, row in importance_df.iterrows():

hatch = '--------' if row['direction'] == 'Negative' else None

ax.barh(

row['feature'],

row['mean_abs_shap'],

color='white',

edgecolor='black',

hatch=hatch,

height=0.7

)

ax.set_yticks(range(len(importance_df)))

ax.set_yticklabels([f.capitalize() for f in importance_df['feature']])

for label in ax.get_yticklabels():

label.set_fontname('Times New Roman')

label.set_fontsize(10)

ax.set_xlabel('Mean Abs SHAP Value', fontname='Times New Roman', fontsize=11)

ax.set_title(f"{group_name} - {target_labels[i]}\nR²={r2:.3f}, RMSE={rmse:.4f}",

fontsize=12, fontweight='bold', fontname='Times New Roman')

ax.grid(False)

if i == 1 and j == 1:

ax.text(

0.95, -0.25,

"Hatched bars indicate negative SHAP contributions",

ha='right', va='center',

fontsize=9, fontname='Times New Roman',

transform=ax.transAxes

)

plt.suptitle("",

fontsize=16, fontweight='bold', fontname='Times New Roman')

plt.show()

# #THE END
