# Supplementary material for: Effectiveness of roadside alcohol testing in reducing fatal accidents and fatal drinking-driving accidents: A multi-city study in China
Source: PLoS One. 2026 Jan 23;21(1):e0338886. doi: 10.1371/journal.pone.0338886 (PMC12829829; doi:10.1371/journal.pone.0338886)
Supplement: Attachment — Submitted filename: Response to Reviewers.docx [file pone.0338886.s002.docx]

PONE-D-25-37604

Effectiveness of Roadside Alcohol Testing in Reducing Fatal Accidents and Fatal Drinking-Driving Accidents: A Multi-City Study in China

PLOS ONE

Please include the following items when submitting your revised manuscript:

- A rebuttal letter that responds to each point raised by the academic editor and reviewer(s). You should upload this letter as a separate file labeled 'Response to Reviewers'.
- A marked-up copy of your manuscript that highlights changes made to the original version. You should upload this as a separate file labeled 'Revised Manuscript with Track Changes'.
- An unmarked version of your revised paper without tracked changes. You should upload this as a separate file labeled 'Manuscript'.

If you would like to make changes to your financial disclosure, please include your updated statement in your cover letter. Guidelines for resubmitting your figure files are available below the reviewer comments at the end of this letter.

Reply

All the required documents are ready for submission. The funding source has been included in the cover letter.

Journal requirements:

When submitting your revision, we need you to address these additional requirements.

1. Please ensure that your manuscript meets PLOS ONE's style requirements, including those for file naming. The PLOS ONE style templates can be found at

<https://journals.plos.org/plosone/s/file?id=wjVg/PLOSOne_formatting_sample_main_body.pdf> and

[https://journals.plos.org/plosone/s/file?id=ba62/PLOSOne_formatting_sample_title_authors_affiliations.pdf](https://track.editorialmanager.com/CL0/https:%2F%2Fjournals.plos.org%2Fplosone%2Fs%2Ffile%3Fid=ba62%2FPLOSOne_formatting_sample_title_authors_affiliations.pdf/1/010f019a28fa7ba3-6ad66d49-4cf9-4cbc-b247-a5b82432dd16-000000/WDZQZWEe66DwBSQ7mU1DoQ9o2iPG3YKbCEBs4jIWRHA=234)

2. We note that Figure 1 in your submission contain [map/satellite] images which may be copyrighted. All PLOS content is published under the Creative Commons Attribution License (CC BY 4.0), which means that the manuscript, images, and Supporting Information files will be freely available online, and any third party is permitted to access, download, copy, distribute, and use these materials in any way, even commercially, with proper attribution. For these reasons, we cannot publish previously copyrighted maps or satellite images created using proprietary data, such as Google software (Google Maps, Street View, and Earth). For more information, see our copyright guidelines: <http://journals.plos.org/plosone/s/licenses-and-copyright>.

 We require you to either (1) present written permission from the copyright holder to publish these figures specifically under the CC BY 4.0 license, or (2) remove the figures from your submission:

 1. You may seek permission from the original copyright holder of Figure 1 Publish the content specifically under the CC BY 4.0 license.

We recommend that you contact the original copyright holder with the Content Permission Form (http://journals.plos.org/plosone/s/file?id=7c09/content-permission-form.pdf) and the following text:

“I request permission for the open-access journal PLOS ONE to publish XXX under the Creative Commons Attribution License (CCAL) CC BY 4.0 (http://creativecommons.org/licenses/by/4.0/). Please be aware that this license allows unrestricted use and distribution, even commercially, by third parties. Please reply and provide explicit written permission to publish XXX under a CC BY license and complete the attached form.”

Please upload the completed Content Permission Form or other proof of granted permissions as an "Other" file with your submission.

In the figure caption of the copyrighted figure, please include the following text: “Reprinted from [ref] under a CC BY license, with permission from [name of publisher], original copyright [original copyright year].”

2. If you are unable to obtain permission from the original copyright holder to publish these figures under the CC BY 4.0 license or if the copyright holder’s requirements are incompatible with the CC BY 4.0 license, please either i) remove the figure or ii) supply a replacement figure that complies with the CC BY 4.0 license. Please check copyright information on all replacement figures and update the figure caption with source information. If applicable, please specify in the figure caption text when a figure is similar but not identical to the original image and is therefore for illustrative purposes only.

The following resources for replacing copyrighted map figures may be helpful:

USGS National Map Viewer (public domain): <http://viewer.nationalmap.gov/viewer/>

The Gateway to Astronaut Photography of Earth (public domain): <http://eol.jsc.nasa.gov/sseop/clickmap/>

Maps at the CIA (public domain): <https://www.cia.gov/library/publications/the-world-factbook/index.html> and <https://www.cia.gov/library/publications/cia-maps-publications/index.html>

NASA Earth Observatory (public domain): <http://earthobservatory.nasa.gov/>

Landsat: <http://landsat.visibleearth.nasa.gov/>

USGS EROS (Earth Resources Observatory and Science (EROS) Center) (public domain): [http://eros.usgs.gov/#](http://eros.usgs.gov/)

Natural Earth (public domain): [http://www.naturalearthdata.com/](http://track.editorialmanager.com/CL0/http:%2F%2Fwww.naturalearthdata.com%2F/1/010f019a28fa7ba3-6ad66d49-4cf9-4cbc-b247-a5b82432dd16-000000/x8yRI_4FTUb44FGXqCLD93TJbv_Xy-48w6gDkdnd6V4=234)

3. If the reviewer comments include a recommendation to cite specific previously published works, please review and evaluate these publications to determine whether they are relevant and should be cited. There is no requirement to cite these works unless the editor has indicated otherwise.

Reply

Thank you for your notice regarding the potential copyright issue for Figure 1.
We confirm that the base map in Figure 1 was obtained from the Standard Map Service System of the Ministry of Natural Resources of the People's Republic of China, which is an official and publicly available government data source. According to the website,

“标准地图依据中国和世界各国国界线画法标准编制而成，可用于新闻宣传用图、书刊报纸插图、广告展示背景图、工艺品设计底图等，也可作为编制公开版地图的参考底图。”

Which can be translated as “The standard maps are compiled based on the drawing standards of national boundaries of China and other countries around the world. They can be used for news and publicity maps, illustrations in books and newspapers, background images for advertisements and exhibitions, design bases for handicrafts, and as reference base maps for producing publicly released maps.”

The dataset is openly accessible through the following official website:
<http://bzdt.ch.mnr.gov.cn/download.html?searchText=GS(2019)1822%EF%BC%89>

We used this publicly available government map as the base layer and overlaid population data derived from the national census to produce Figure 1. The final figure is an original composite created by the authors, not a reproduction of any copyrighted or proprietary map (such as Google Maps, Street View, or Earth).
Therefore, there is no copyright infringement concern, and the figure fully complies with the CC BY 4.0 license requirements for open-access publication.

In the revised manuscript, we have clarified this in the figure caption as follows:
Fig. 1. Study regions and city population. Downloaded from the Standard Map Service System of the Ministry of Natural Resources of the People's Republic of China (http://bzdt.ch.mnr.gov.cn/). The base map was freely provided for public use and modified by the authors to include population data from the national census.

Additional Editor Comments (if provided):


Reviewers' comments:

Reviewer's Responses to Questions

**Comments to the Author**

1. Is the manuscript technically sound, and do the data support the conclusions?

The manuscript must describe a technically sound piece of scientific research with data that supports the conclusions. Experiments must have been conducted rigorously, with appropriate controls, replication, and sample sizes. The conclusions must be drawn appropriately based on the data presented.

Reviewer #1: Yes

Reviewer #2: Yes

2. Has the statistical analysis been performed appropriately and rigorously?

Reviewer #1: Yes

Reviewer #2: Yes

3. Have the authors made all data underlying the findings in their manuscript fully available?

The [PLOS Data policy](http://track.editorialmanager.com/CL0/http:%2F%2Fwww.plosone.org%2Fstatic%2Fpolicies.action%23sharing/1/010f019a28fa7ba3-6ad66d49-4cf9-4cbc-b247-a5b82432dd16-000000/hUU7qdeMqyfU97sZU38boREVagGy_B16RuU4ar_vPjI=234) requires authors to make all data underlying the findings described in their manuscript fully available without restriction, with rare exception (please refer to the Data Availability Statement in the manuscript PDF file). The data should be provided as part of the manuscript or its supporting information, or deposited to a public repository. For example, in addition to summary statistics, the data points behind means, medians and variance measures should be available. If there are restrictions on publicly sharing data—e.g. participant privacy or use of data from a third party—those must be specified.

Reviewer #1: Yes

Reviewer #2: No

Reply

The original data can be accessed from the following public source:

Hu D, Chen X, Liu L. (2024). *Full judgment document data for intelligent trial assistance* [面向智能审判辅助的全量裁判文书数据]. *National Big Data Service Platform* [全国数据服务平台]. Available from: <https://www.nbsdc.cn/general/dataDetail?id=66606e34195d266d328f3ca0&type=1>

In addition, the codes used for data mining and the datasets applied in the VAR/IRF models and XGBoost with SHAP analyses have been included as supplementary files with this submission. These materials ensure that all analyses can be replicated and verified in accordance with PLOS ONE’s data availability policy.

4. Is the manuscript presented in an intelligible fashion and written in standard English?

PLOS ONE does not copyedit accepted manuscripts, so the language in submitted articles must be clear, correct, and unambiguous. Any typographical or grammatical errors should be corrected at revision, so please note any specific errors here.

Reviewer #1: Yes

Reviewer #2: Yes

5. Review Comments to the Author

Please use the space provided to explain your answers to the questions above. You may also include additional comments for the author, including concerns about dual publication, research ethics, or publication ethics. (Please upload your review as an attachment if it exceeds 20,000 characters)

Reviewer #1: In the manuscript titled “Effectiveness of Roadside Alcohol Testing in Reducing Fatal Accidents and Fatal Drinking-Driving Accidents: A Multi-City Study in China”, the authors analyzed a large-scale dataset (248 cities, 2014–2020) and applied both VAR/IRF models and XGBoost with SHAP analysis to investigate the dynamic effects of roadside alcohol testing on traffic mortality. This study contains interesting findings and provides valuable evidence for understanding the deterrent effects of DUI enforcement, especially the heterogeneous effects between small and large cities.
However, the manuscript also presents some weaknesses in terms of structure, literature review depth, and data/method transparency. Therefore, major revision has to be done before this manuscript could be accepted for publication in PLOS ONE.

1. The transition between literature review and research gap is not sufficiently clear. The authors should streamline this part and highlight the novelty of their study more explicitly.

2. The review cites many studies, but some are descriptive rather than analytical. Please strengthen the discussion with recent works on machine learning applications in traffic safety and international comparisons of enforcement strategies. I suggest the author integrate these articles:Sight distance analysis of vehicles with driving automation on horizontal curves of as-built highway tunnels; A system theory based accident analysis model: STAMP-fuzzy DEMATEL; Temporal heterogeneity in traffic crash delays: causal inference from multi-scale time factors and sample-wise structural decomposition. So, I believe this research will be greatly improved.

Reply

To add the transition between literature review and research gap, we have read the three articles and added a new paragraph to increase the smooth transition:

“Previous studies have primarily relied on traditional time-series approaches, such as interrupted time series analysis, which can only compare changes in mortality trends between two time periods and are therefore typically applied to evaluate pre- and post-legislation effects. In contrast, mixed-effects regression models can accommodate hierarchical structures and control for observed confounders [14]; however, they generally impose a linearity assumption that may not adequately capture the complex relationship between enforcement intensity and traffic mortality. Moreover, traffic accident data often exhibit weak temporal dependence and non-stationary characteristics, making traditional time-series modeling challenging [17]. Therefore, it is necessary to complement conventional regression approaches with machine learning models that relax the linearity assumption, enabling a more flexible assessment of enforcement impacts while more effectively addressing confounding factors [18]. In addition, the use of SHapley Additive exPlanations (SHAP) values provides an interpretable framework to quantify the contribution of enforcement, among a set of potential confounders, to variations in mortality rates [19].”

3. The reliance on court judgment documents introduces potential selection bias (e.g., only cases with on-site deaths and surviving offenders are included). This limitation should be emphasized more clearly in the methodology and discussion.

Reply

In the first paragraph of the Discussion, we have stated that:

“It is important to note that court system data may underreport traffic-related mortalities in China, as court records typically include only cases in which the victim died at the scene and the offender survived. In contrast, the health department is able to capture fatalities that occur after prolonged hospitalization, as well as deaths of both victims and offenders [23]. Despite this potential underreporting, the number of cases retrieved from court documents remains substantial, underscoring the importance of investigating whether increased frequency of roadside checks contributes to a reduction in traffic-related fatalities.”

4. Only XGBoost was employed in the machine learning part. Please justify why no comparison with other algorithms (e.g., Random Forest, LSTM) was attempted. This would enhance methodological robustness.

Reply

We appreciate the reviewer’s insightful comment. The three papers mentioned indeed focused mainly on engineering applications, where machine learning models are optimized for predictive accuracy to enhance traffic or engineering safety. In contrast, the current study emphasizes the policy dimension. Specifically, the effectiveness of law enforcement policies. Therefore, our modeling objective differs from that of engineering-oriented research.

Unlike engineering studies that pursue highly precise predictive performance, the mathematical modeling of law enforcement policy does not necessarily benefit from such optimization. Even if a model achieves the best performance on the current dataset, it may not generalize well under different political systems and cultural contexts, resulting in potential overfitting. Consequently, in this study we selected the XGBoost model from three candidate algorithms through a grid search for optimal hyperparameters, without exhaustively testing all machine learning methods or tuning a larger set of parameters. This decision reflects our intention to balance model interpretability and generalizability rather than to over-optimize performance on a single dataset.

In the Method part, we added:

“To account for confounding effects that traditional time-series methods cannot adequately address, we employed Gradient Boosted Regression Trees (GBRT) implemented via the XGBoost library. Before finalizing the model, we compared three candidate algorithms, namely Random Forest, Support Vector Regression, and XGBoost. A grid search procedure was used to identify the optimal set of hyperparameters for each model. Based on both predictive performance and interpretability considerations, XGBoost was selected as the final model because it provided an appropriate balance between coefficient of determination (R²) and root mean squared error (RMSE).”

In the limitation, we emphasized that:

“This study is based on data from China, and the results may not be directly generalizable to other countries or regions. Significant differences in enforcement culture, social norms, and DUI laws across countries could impact the generalizability of policy effects.”

5. Some expressions could be more precise. For example, “mortality plateau once roadside check rates reach approximately 0.002” could be rephrased in more formal academic language.

Reply

We have rephrased the expression as:

“Marginal effect analysis revealed a pattern of diminishing returns in large cities, where reductions in mortality began to stabilize once roadside check rates reached approximately 0.002 per 100,000 people per month.”

6. Some references are incomplete or not in standard PLOS ONE format (e.g., [7]). Please revise and ensure DOI information is provided where available.

Reply

We have updated the reference to:

“Malta, D. C., Morais, O. L. D., Cardoso, L. S. D. M., Veloso, G. A., Andrade, F. M. D. D., Vasconcelos, A. M. N., ... & Naghavi, M. (2022). Road traffic injuries and deaths and the achievement of UN Sustainable Development Goals in Brazil: results from the Global Burden of Disease Study, 1990 to 2019. Revista da Sociedade Brasileira de Medicina Tropical, 55(suppl 1), e0261-2021. <https://doi.org/10.1590/0037-8682-0261-2021> ”

The corresponding sentences in the Introduction have been revised as follows:

“In 2008, Brazil further amended the law to adopt a zero-tolerance policy for DUI, setting the legal BAC limit at 20 mg/100 ml [8]. However, while the mortality rates for pedestrians decreased, those for motorcyclists and cyclists increased [7].”

As to the three Chinese language references, no DOI number is provided, and thus absent.

7. Please add a section on future research directions, such as integrating multi-source data (e.g., hospital, police, insurance) or applying causal inference methods (DID, IV).

Reply

In the Conclusion, we added the following statement:

“Future studies should merge court records with hospital trauma registries, police patrol logs, and insurance telematics to construct kilometer-based enforcement measures. They could then exploit staggered municipal rollouts or randomized checkpoints as instrumental variables or Difference-in-Differences (DID) shocks to verify whether the observed eight-month mortality reduction represents a true causal deterrent effect rather than reactive policing or unobserved confounding shocks.”

Reviewer #2: This manuscript presents a valuable multi-city study in China investigating the effectiveness of roadside alcohol testing in reducing traffic fatalities, using a combination of Vector Autoregression (VAR) for temporal dynamics and XGBoost for nonlinear pattern analysis. The topic of this paper is very interesting. However, several revisions are needed to strengthen the manuscript’s rigor, clarity, and compliance with PLOS ONE standards.
1. Inconsistencies and gaps mar the reference section. First, Jomar et al. (2018) is labeled "Unpublished or source unspecified," which violates PLOS ONE’s preference for peer-reviewed or formally archived sources.

Reply

We have updated the reference to:

“Malta, D. C., Morais, O. L. D., Cardoso, L. S. D. M., Veloso, G. A., Andrade, F. M. D. D., Vasconcelos, A. M. N., ... & Naghavi, M. (2022). Road traffic injuries and deaths and the achievement of UN Sustainable Development Goals in Brazil: results from the Global Burden of Disease Study, 1990 to 2019. Revista da Sociedade Brasileira de Medicina Tropical, 55(suppl 1), e0261-2021. <https://doi.org/10.1590/0037-8682-0261-2021> ”

The corresponding sentences in the Introduction have been revised as follows:

“In 2008, Brazil further amended the law to adopt a zero-tolerance policy for DUI, setting the legal BAC limit at 20 mg/100 ml [8]. However, while the mortality rates for pedestrians decreased, those for motorcyclists and cyclists increased [7].”

2. Some Chinese reference appears.

Reply

The three Chinese references have been intentionally retained because each serves a distinct and essential purpose in the study. Specifically, *Zhang (2024)* provides critical contextual insights into the legal effectiveness of drunk-driving enforcement, which offers an important comparative reference for interpreting our results. *Hu et al. (2024)* describes the national-level open database from which our data were obtained, while *Song (2023)* discusses issues of data reliability and security in judgment document disclosure. Together, these sources contribute directly to the transparency and contextual validity of our research.

We acknowledge that these publications do not have DOI numbers, as they are part of Chinese academic journals or official national data platforms that have not yet assigned DOIs. In accordance with PLOS ONE referencing standards, we have retained these entries with full citation details to ensure traceability and scholarly completeness.

3. Though the study claims "all data are fully available without restriction," the Data Availability Statement lacks actionable details.

Reply

The original data can be accessed from the following public source:

Hu D, Chen X, Liu L. (2024). *Full judgment document data for intelligent trial assistance* [面向智能审判辅助的全量裁判文书数据]. *National Big Data Service Platform* [全国数据服务平台]. Available from: <https://www.nbsdc.cn/general/dataDetail?id=66606e34195d266d328f3ca0&type=1>

In addition, the codes used for data mining and the datasets applied in the VAR/IRF models and XGBoost with SHAP analyses have been included as supplementary files with this submission. These materials ensure that all analyses can be replicated and verified in accordance with PLOS ONE’s data availability policy.

4. Several figure-related issues hinder interpretability and are very vague.

Reply

We have carefully revised all five figures to meet PLOS ONE’s requirements. In addition, we have enlarged the font sizes in the figures to enhance clarity and make them easier to interpret. We hope these changes address the concerns raised.

5.The study’s conclusions would benefit from additional robustness checks. For the VAR model, only AIC is used to select lag order—no sensitivity analysis is provided to confirm if results hold with different lags. For XGBoost, parameters are not tuned.

Reply

Robustness Checks:

In the Methods, we added:

“To ensure robustness, additional estimations were conducted using alternative lag lengths ($p=4 and 8$).”

In the Results, we added:

“To test robustness, the VAR models were re-estimated using lag orders of 4 and 8. The impulse response functions exhibit highly consistent patterns across specifications. The peak responses occur around the first month in all cases, and the magnitude and direction of the effects change only marginally. These results confirm that the estimated IRFs are robust to alternative lag selections.”

Selection of Machine Learning Models and Hyperparameter Tuning:

In the Methods, we added:

“Before finalizing the model, we compared three candidate algorithms, namely Random Forest, Support Vector Regression, and XGBoost. A grid search procedure was used to identify the optimal set of hyperparameters for each model. Based on both predictive performance and interpretability considerations, XGBoost was selected as the final model because it provided an appropriate balance between coefficient of determination (R²) and root mean squared error (RMSE).”

Moreover, as noted by the first reviewer, exhaustive hyperparameter tuning is not essential in this context. Since the study aims to model policy effects rather than maximize predictive accuracy, excessive tuning may lead to overfitting and reduce generalizability across different political and cultural contexts. Therefore, we adopted a balanced approach: grid search was used to determine reasonable hyperparameters, and XGBoost was selected among three well-established algorithms without over-optimizing model performance. This approach ensures an appropriate trade-off between interpretability and generalizability.
